# Embedded Printing of Integrated Quantum Dot Waveguide Deformation Sensors

**DOI:** 10.3390/s26041160

**Published:** 2026-02-11

**Authors:** Tobias Biermann, Lennart Mesecke, Simon Teves, Gerrit Eckert, Ole Hill, Ivo Ziesche, Alexander Wolf, Roland Lachmayer

**Affiliations:** 1 Institute of Product Development, Leibniz University Hannover, An der Universität 1, 30823 Garbsen, Germanyteves@ipeg.uni-hannover.de (S.T.);; 2 Cluster of Excellence PhoenixD, Leibniz University Hannover, Welfengarten 1A, 30167 Hannover, Germany; 3 Institute of Physical Chemistry and Electrochemistry, Leibniz University Hannover, Callinstraße 3-3a, 30167 Hannover, Germany; 4 Laser Zentrum Hannover e.V., Hollerithallee 8, 30419 Hannover, Germany; 5 Deutsches Elektronen-Synchrotron, Platanenallee 6, 15738 Zeuthen, Germany; 6 Deutsches Zentrum für Astrophysik, Postplatz 1, 02826 Görlitz, Germany

**Keywords:** optical deformation measurement, quantum dots, fluorescence, embedded printing, additive manufacturing, soft robotics, condition monitoring

## Abstract

We present an optical deformation sensor additively manufactured via an embedded printing process that enables the direct integration of colloidal quantum dots into multimode silicone (PDMS) waveguides. The sensor consists of two parallel waveguide strands, one of which is locally functionalized with CdSe/CdS quantum dots serving as fluorescent emitters. When narrow-band UV light at 405 nm is coupled into the non-functionalized strand, structural deformation alters the conditions of total internal reflection, thereby changing the optical interaction between both strands. This leads to a deformation-dependent variation in the fluorescence shift-affected intensity ratio, which serves as a self-referenced signal for angle determination. Using ratiometric evaluation, angular deflections of up to 9.5° are detected with a resolution below 1° (2σ confidence), representing the performance of an initial functional prototype. The embedded printing process allows the voxel-wise adjustment of the material composition within a viscoplastic support medium and thus the spatially resolved integration of quantum dot-functionalized silicone. Attenuation losses of 0.81±0.02dB/cm at 625 nm confirm the optical suitability of the printed waveguides. This approach combines optical sensing and structural flexibility within a single manufacturing step and establishes a pathway toward fully integratable deformation-sensing elements for soft robotic and wearable systems.

## 1. Introduction

Deformation measurement on the surfaces of objects is important for many applications, such as condition monitoring [1], providing feedback for soft robotic movements [2,3,4], enabling wearable strain sensors [5] and advancing health monitoring systems [6]. Traditionally, electrical and optical sensors have been used for this purpose [5,7]. The advantages of optical systems lie in their high electromagnetic compatibility and their achievable fast measurement times. For deformation detection, optical sensors such as remote imaging systems and fiber-based sensors are employed, with the latter being directly attached to or embedded in the measurement object.

In conventional optical fibers, strain measurement can be realized using fiber Bragg gratings (FBGs) [8]. These gratings, inscribed periodically in the fiber core, reflect a distinct wavelength that shifts under axial elongation. While FBGs in glass or polymer fibers allow the highly resolved detection of axial strain, they require complex spectral evaluation [8,9].

Other fiber-based approaches exploit changes in total internal reflection and absorption. In multimode fibers, bending increases the number of outcoupled rays, leading to a measurable reduction in the transmitted intensity [10,11]. Krauss et al. demonstrated a stretchable optical waveguide sensor with a semi-divided optical core, enabling the simultaneous detection of the deformation amplitude and direction through the differential light intensity [12]. Trunin et al. developed a soft optical waveguide sensor that utilizes the outcoupling of rays on a structured surface while bending [13]. Bai et al. introduced a stretchable distributed fiber-optic sensor composed of elastomeric lightguides with chromatic patterns. The system is integrated into a wireless glove and detects the position, magnitude and mode of mechanical deformation such as stretching, bending or pressing [14]. Wolf and Wüllner et al. presented a prismatic optical fiber for torsion measurement, detecting the angular deflection and transmitted intensity changes of a guided laser beam [15,16]. Chen et al. developed a dual-core stretchable polymer optical fiber composed of a circular PDMS core and a flat fluorinated PMMA core. Their system operates based on optical intensity modulation, allowing the simultaneous detection of the applied force magnitude and direction [17].

Planar optical structures have also been explored for deformation sensing, where the coupling efficiency between two adjacent light-guiding elements changes under strain [1]. Such structures can be fabricated by flexographic printing on thin polymer foils [18,19]. Furthermore, doping waveguides with fluorescent materials enables optical strain sensing. Guo et al. developed a flexible optical strain sensor based on silicone fibers doped with Rhodamine dye, where elongation induces a strain-dependent absorption change [20]. Li et al. reported a fluorescence-based pressure sensor utilizing a PDMS liquid-core optical fiber in which the deformation of the cladding alters the total internal reflection, thereby modulating the emitted fluorescence [21].

Despite these developments, current optical strain-sensing approaches face challenges, such as complex spectral evaluation, restriction to uniaxial deformation states, environmental susceptibility and long-term degradation. In applications involving highly deformable materials, such as soft robotics, conventional strain gauges or rigid optical fibers cannot follow large deformations or be seamlessly integrated into soft structures. These limitations motivate the development of optical sensor concepts based on flexible and transparent polymers that combine mechanical compliance with optical functionality.

Additive manufacturing offers a promising pathway toward the direct integration of deformation sensing, since soft robotic structures are often fabricated with additive manufacturing techniques utilizing elastic materials such as silicone [22,23,24]. Maiwald et al. demonstrated the layer-wise printing of metallic strain gauges on polymer substrates, highlighting the feasibility of integrating sensor structures within flexible systems; however, their approach relies on electrical signal transduction rather than optical principles [25]. Muth et al. extended this concept through the three-dimensional embedded printing of conductive elastomer networks, enabling the monolithic integration of soft strain sensors [7]. These studies emphasize the potential of additive manufacturing for the voxel-wise control of material compositions within soft environments.

Building on these foundations, additive manufacturing has been successfully applied to the fabrication of optical components such as lenses and waveguides [26,27,28]. Embedded printing, in particular, enables the voxel-wise placement of optically functional materials within soft, transparent silicone matrices [29], as well as the printing of soft robotic structures [30,31]. This additive manufacturing process serves as the technological basis for the optical deformation sensor presented in this work.

Our work presents an optical deformation sensor fabricated via embedded printing, where the primary contribution is the manufacturing-enabled, voxel-wise integration of colloidal quantum dots into flexible, transparent silicone waveguides, enabling a spatially confined sensing function that is not accessible with conventional waveguide fabrication routes. The novel sensor architecture consists of two parallel multimode silicone waveguides, one of which is locally functionalized with CdSe/CdS quantum dots, forming a dual-core structure. When narrow-band UV light is coupled into the system, structural deformation alters the geometrical conditions of light propagation and total internal reflection, thereby modifying the interaction of the guided excitation light with the functionalized strand. This leads to a deformation-dependent change in the ratio of non-converted excitation light to quantum dot fluorescence. While ratiometric signal evaluation is a well-established concept, its implementation here is enabled by the localized, in-process functionalization of one waveguide strand within an otherwise homogeneous printed structure. By monitoring this fluorescence-to-excitation intensity ratio, angular deformations can be detected, demonstrating embedded printing as a key enabler for integrated optical deformation sensing for flexible structures.

## 2. Materials and Methods

### 2.1. Measurement Principles and Sensor Concept

**Figure 1:**
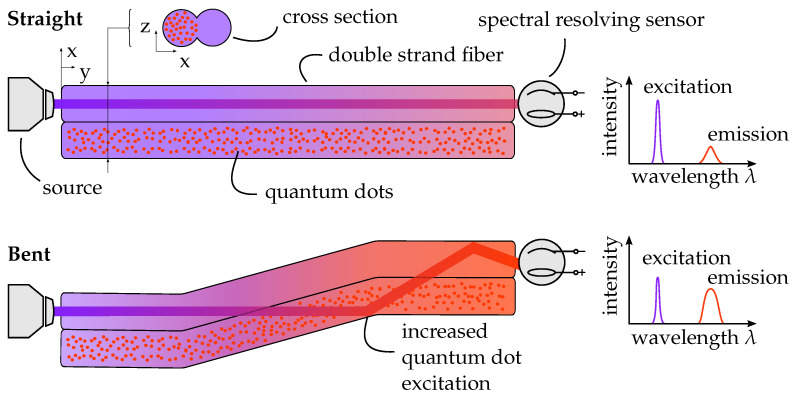
Schematic representation of the sensor concept. Two parallel multimode silicone waveguides are used, one of which is locally functionalized with colloidally dispersed CdSe/CdS quantum dots. Bending of the waveguide changes the internal intensity distribution and thereby the fluorescence shift-affected intensity ratio detected at the distal end.

The proposed deformation sensor operates on optical principles based on the total internal reflection within flexible, multimode silicone waveguides. The conceptual structure is shown in Figure 1.

Two parallel strands of optically transparent silicone form the waveguide system. One strand serves as a passive reference, while the second is locally functionalized by the in-process incorporation of colloidally dispersed CdSe/CdS quantum dots. These nanoparticles act as fluorescent emitters when excited by the excitation wavelength.

Narrow-band UV excitation light is coupled into the end facet of the sensor. The emission cone of the light source is chosen such that the guided rays satisfy the condition for total internal reflection at the silicone–air interface. Depending on the numerical aperture and cross-sectional geometry of the waveguide, an intensity distribution I(x,y,z) develops along the propagation axis *z*. In the straight, undeformed state ($\beta =0^{\circ }$), this distribution is stationary and defines the local excitation intensity within the functionalized region.

Upon bending of the waveguide by an angle β, the propagation angles of individual modes are modified, leading to the redistribution of the optical intensity within the cross-section. As a consequence, the number of photons interacting with the quantum dots changes. The embedded quantum dots absorb part of the short-wavelength excitation flux $\Phi_{\mathrm{exc}}$ and re-emit light at a longer wavelength due to the Stokes shift. The resulting long-wavelength converted flux $\Phi_{\mathrm{conv}}$ therefore depends on the local short-wavelength intensity and, indirectly, on the applied deformation β (see Figure 4).

Both spectral components, the remaining short-wavelength transmitted flux $\Phi_{\mathrm{trans}}$ and the long-wavelength converted flux $\Phi_{\mathrm{conv}}$, propagate to the output facet. A wavelength-selective detector separates and records both contributions. The deformation state can thus be quantified by the ratio

$$R=\frac{\Phi_{\mathrm{conv}}}{\Phi_{\mathrm{trans}}}.$$

which increases with the fraction of guided light converted by the quantum dots.

For purposes of compensating for variations in source intensity, coupling efficiency and optical losses, the normalized peak intensity ratio $R_{norm}$ is introduced. It is defined by dividing R(β) by the peak intensity ratio in the undeflected $R(0^{\circ })$ state:

$$R_{norm}\left(\beta \right)=\frac{R(\beta )}{R(0^{\circ })}.$$

### 2.2. Manufacturing Process: Embedded Printing

**Figure 2:**
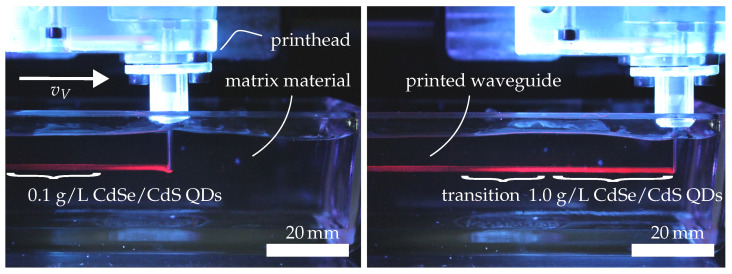
Visualization of a material transition in the active-mixing printhead during the embedded printing process. The volumetric mixing ratio of component A containing colloidally dispersed CdSe/CdS quantum dots is changed to result in a CdSe/CdS quantum dot change from 0.1 wt.% to 1.0 wt.%.

The sensor structure consisting of two parallel silicone waveguides is fabricated using an embedded printing process, in which a two-component silicone is extruded into a yield stress, shear-thinning support medium [7,30,31,32]. This process allows the deposition of slowly curing, highly transparent silicones without the need for solid support structures. During printing, the nozzle is translated within the supporting matrix and deposits the structural material along predefined tool paths. The surrounding matrix prevents the gravitational collapse of the uncured strand by providing yield stress-based confinement. After curing, the matrix material is removed, leaving a free-standing silicone structure. By employing an active mixing, multicomponent printhead, the local composition of the extruded material can be adjusted during continuous extrusion. This enables the selective integration of colloidally dispersed nanoparticles into defined regions of the printed waveguide, providing voxel-wise control over the optical functionality. A material transition between quantum dot concentrations from low to high is shown in Figure 2.

#### 2.2.1. Custom Printer Setup

The embedded printing process is performed on a custom-built system based on a three-axis Cartesian gantry platform (Snapmaker 2.0, Snapmaker Inc., Shenzhen, China). Material delivery is realized using a multicomponent extrusion unit (Viscotec ViproHEAD 3/3, ViscoTec Pumpen- u. Dosiertechnik GmbH, Töging am Inn, Germany), which is extended by a custom-developed printhead for embedded printing applications. The printhead introduced by Teves et al. integrates a hydrodynamically balanced switching unit and an active inline mixing system driven by a brushed DC motor (7V brushed motor, Conrad Electronic SE, Hirschau, Germany), enabling minimized transition volumes between different material compositions while ensuring the homogeneous mixing of the reactive silicone components prior to extrusion [29]. Extrusion is carried out through a replaceable nozzle (standard 1/4" luer-lock 16-gauge dispensing tips, Vieweg GmbH, Kranzberg, Germany). Motion control, material extrusion and active mixing are synchronized using a Bigtreetech Octopus V1.1 control board (BigTreeTech, Shenzhen, China) running Marlin v2.1.3 firmware in combination with TMC2209 V1.2 stepper drivers (Trinamic Motion Control GmbH, Hamburg, Germany).

#### 2.2.2. Optical Carrier Material: Dowsil™ EI-1184

As an optical carrier material, a room-temperature vulcanizing two-component (RTV-2) silicone (Dowsil™ EI-1184, Dow Inc., Midland, TX, USA) is used. After the mixing of the base and curing agent at a ratio of 1:1, the material cures within 4 h at 25 °C or 5 min at 80 °C. Once cured, it forms a transparent elastomer with a refractive index of n=1.42 and transmittance of approximately 94 % (10 mm sample) in the visible range, with the stated value referring to Fresnel reflection-corrected transmission. The low modulus and high flexibility of EI-1184 further allow large elastic deformations [29].

#### 2.2.3. Quantum Dots: CdSe/CdS

The functionalization of the waveguide relies on semiconductor quantum dots with a CdSe/CdS core–shell structure, synthesized in-house at the Institute of Physical Chemistry and Electrochemistry of the Leibniz University Hannover, according to [29,33]. The CdSe cores have a diameter of approximately 4–5 nm, surrounded by a CdS shell of about 1–4 nm thickness. The resulting particles exhibit an emission maximum near 615–650 nm with a photoluminescence quantum yield of 60–80 %. The core–shell architecture passivates non-radiative surface states and ensures high fluorescence efficiency. The quantum dots are synthesized via a two-step hot injection process and subsequently dispersed colloidally in non-polar solvents (e.g., toluene or hexane). Prior to printing, they are blended into one component of the EI-1184 silicone precursor system to obtain a stable dispersion. Ligand exchange ensures chemical compatibility after curing. Furthermore, the immobilization of the CdSe/CdS quantum dots within the crosslinked silicone matrix has been shown to effectively suppress cadmium release, enabling safe handling under standard laboratory conditions, as demonstrated by Eckert et al. [33]. Temperature-dependent photoluminescence studies on CdSe-based quantum dot clusters indicate only small and reversible changes in emission under moderate temperature variations [34]. Furthermore, CdSe/CdS core–shell quantum dots are known for their high photostability under continuous optical excitation, owing to the effective passivation by the shell [35].

#### 2.2.4. Matrix Material: Copsil^®^ Add-Gel

As a supporting matrix for the embedded printing process, viscoplastic materials with a pronounced yield point are utilized [32,36,37]. Here, the viscoplastic gel Copsil^®^ Add-Gel (COP Chimie, Saints-Fons, France) is used as the supporting medium. This material behaves as a Herschel–Bulkley fluid with pronounced yield stress, allowing the nozzle to move through the gel while the surrounding medium immediately resolidifies behind it [38]. The matrix stabilizes the deposited silicone strands during curing and prevents sedimentation or deformation [39,40]. After the crosslinking of the structural material, the gel can be removed by washing with distilled water, leaving optically clear, free-standing waveguide structures.

#### 2.2.5. Sensor Sample Preparation

**Figure 3:**
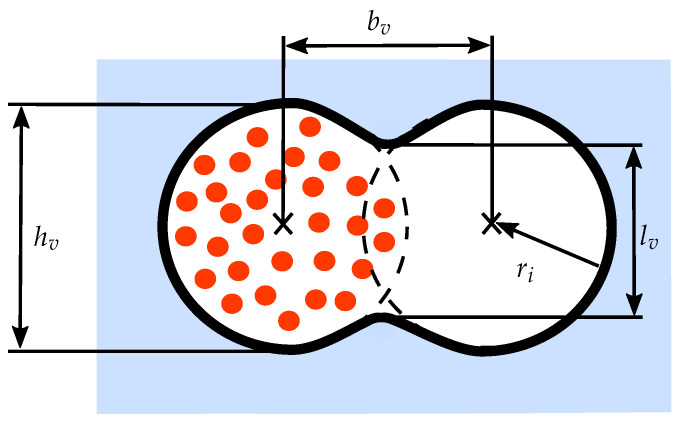
Geometric parameters describing the overlap between two adjacent printed silicone strands. The red dots in the left strand symbolize the colloidally dispersed quantum dots. The strand centers are separated by the distance $b_{v}$, each with radius $r_{i}$. The fused region is characterized by the strand height $h_{v}$ and the bonding length $l_{v}$.

For the fabrication of the deformation sensor samples, an overlap ratio of

$$U\left(b_{v}\right)=\left(1-\frac{b_{v}}{2r_{i}}\right)\times 100\;\%=20\;\%$$

and a fused cross-section with a bonding length of $l_{v}\approx 1000$ μm according to Figure 3 were defined. Printing was carried out using a nozzle with an inner diameter of $d_{i}=2r_{i}=1.19\;\mathrm{mm}$, which also defined the resulting strand diameter and thus ensured stable extrusion within the matrix. The process parameters for the embedded printing process were optimized to eliminate effects such as cavities, nozzle wetting, constrictions or material ruptures, as well as to ensure continuous material fusion of the parallel strands [31,32,37,39,40,41,42,43].

The CdSe/CdS functional material concentration in the silicone was set to 0.5 g/L. This concentration represents a suitable compromise between optical attenuation and the conversion of guided excitation light into fluorescence, yielding a well-defined deformation-dependent signal without excessive losses [29,33]. The spatial distribution of colloidally dispersed CdSe/CdS quantum dots within printed silicone was characterized by Hill et al. and Teves et al. using scanning laser optical tomography (SLOT) [29,44]. Single printed strands were optically sectioned in a fluorescence setup to reconstruct volumetric intensity maps. Mixing quality was quantified via local intensity variations across multiple cross-sections, expressed by the coefficient of variation [29]. As successful mixing had already been demonstrated for the same printing setup, no further measurements were conducted here [29,44].

During printing, the sensor structures were produced in two consecutive steps. First, the strand containing the functionalized silicone was printed to form the fluorescent sensing section. Subsequently, the material feed was switched to pure silicone, and the system was purged with QD-free silicone until only particle-free material was extruded, enabling the rapid continuation of the printing process and the formation of an integrated dual-strand sensor. As silicone crosslinking progresses during printing, this transition step is time-critical; therefore, a print speed of $v_{V}$ = 30 mm/s was selected to ensure reliable bonding between both sections. The selected print speed represents a robust operating point identified through empirical process optimization, for which the temporal offset between depositing the two strands at the same longitudinal position remained below one minute, thereby preventing premature crosslinking and ensuring reliable interfacial adhesion. In total, ten sensor samples were fabricated. From these, three samples were selected for optical characterization based on fabrication-related quality criteria. Specifically, selection was based on continuous and well-defined strand fusion at the interface between the two parallel waveguides and the absence of visible fabrication defects such as inclusions, air bubbles or local discontinuities along the sensing region. These criteria were examined using a Keyence VHX-7000 digital microscope (Keyence Corporation, Osaka, Japan). No selection was performed based on the sensor response or performance metrics.

### 2.3. Characterization Methods

#### 2.3.1. Attenuation Measurement

Attenuation was determined on twenty non-functionalized single-strand waveguides using the cut-back method. These values therefore represent the loss characteristics of the silicone waveguides without quantum dot-induced contributions. Light from fiber-coupled LEDs was butt-coupled into the waveguide via a Thorlabs M45L01 400 µm SMA fiber (NA = 0.5, Thorlabs Inc., Newton, MA, USA). For excitation at 405 nm, a Thorlabs M405FP1 LED (Thorlabs Inc., Newton, MA, USA) with measured output power of 9.0 mW at the fiber end was employed; for 625 nm measurements, a Thorlabs M625F2 LED with 29.9 mW output power at the fiber end was employed. The transmitted power was measured using a Thorlabs S140C integrating-sphere photodiode power meter (Thorlabs Inc., Newton, MA, USA) in combination with a Thorlabs FP1500ERT 1500 µm SMA collection fiber (NA = 0.5, Thorlabs Inc., Newton, MA, USA). After the initial measurement at a waveguide length of 8 cm, the strand was shortened to 6 cm and 5 cm, and the procedure was repeated to obtain the attenuation coefficient α from the slope of the logarithmic power-length relation. Measurements were carried out at 405 nm and 625 nm to represent the excitation and emission regions relevant to the sensor. To analyze bending-dependent losses, the waveguides were mounted on a rotary stage and bent stepwise between 0° and 50° in 5° increments, with the transmitted intensity recorded at each position.

#### 2.3.2. Deformation Experiments

**Figure 4:**
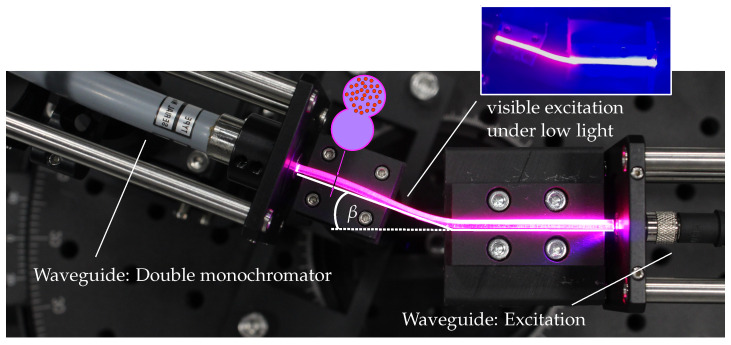
Experimental setup for optical deformation measurements including positioning of the waveguide cross section (cf. Figure 3). Excitation light at 405 nm is coupled into the non-functionalized waveguide strand, while the emitted and transmitted light is directed to the double monochromator for spectral analysis. The inset shows the visibly excited section of the functionalized strand under low ambient light.

To characterize the optical response under mechanical deformation, three dual-strand sensors were fabricated according to Section 2.2. Excitation light at 405 nm was coupled into the non-functionalized strand using a fiber-coupled Thorlabs M405FP1 LED (24.3 mW output, Thorlabs Inc., Newton, MA, USA) connected via a Thorlabs M45L01 400 µm SMA fiber (NA = 0.5, Thorlabs Inc., Newton, MA, USA). The fiber-to-sensor coupling was aligned using a three-axis micropositioning unit to ensure coupling exclusively into the non-functionalized strand. The emitted and transmitted light at the opposite end of the sensor was collected with a Thorlabs FP1500ERT 1500 µm SMA fiber (Thorlabs Inc., Newton, MA, USA) and spectrally analyzed using a Bentham IDR-300 PSL double monochromator (Bentham Instruments Ltd., Reading, UK). The experimental setup is shown in Figure 4.

In the first test, the selected sensors and one reference sensor were investigated. For the reference sensor, both strands were fabricated with CdSe/CdS-functionalized silicone to verify that no measurable optical effect occurred when both sides were emissive. The initial investigation was carried out in 10° increments within a deflection range of $\beta =-40^{\circ }$ to $+40^{\circ }$, where negative values represent bending toward the functionalized strand.

In the main study, the sensors were examined in 3° steps for β=0–$30^{\circ }$ to further refine the measurement range. For one sensor, three repeated measurements were performed in 3° steps over β=0–$15^{\circ }$, with the repositioning of the sensor strands between each run to evaluate measurement repeatability. Subsequently, the same sensor was analyzed in 0.5° increments over β=0–$10^{\circ }$, with the entire measurement range repeated five times to determine the angular resolution and standard deviation, again repositioning after each measurement.

For each deflection, the transmitted flux $\Phi_{\mathrm{trans}}$ and the converted fluorescence flux $\Phi_{\mathrm{conv}}$ were recorded, and the normalized ratio $\Phi_{\mathrm{conv}}/\Phi_{\mathrm{trans}}$ relative to the undeformed state was evaluated. From the standard deviation of the measured values, the achievable angular resolution of the deformation sensor was determined.

## 3. Results

### 3.1. Attenuation Measurements

**Figure 5:**
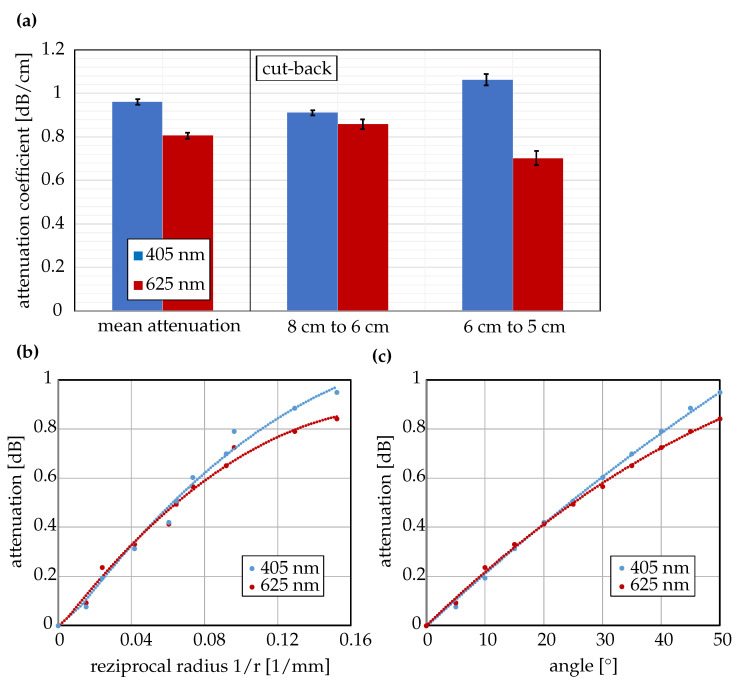
Attenuation measurements of printed waveguides. (**a**) Mean attenuation and results from cut-back measurements at 405 nm and 625 nm. (**b**) Attenuation as a function of the reciprocal bending radius. (**c**) Attenuation as a function of bending angle β. Radius and angle were obtained simultaneously; thus, the data in (**b**,**c**) correspond to the same underlying measurement points.

The attenuation characteristics of the printed waveguides were evaluated at wavelengths of 405 nm and 625 nm using the cut-back method described in Section 2.3. The resulting attenuation coefficients, shown in Figure 5, were determined as $\alpha_{405\;\mathrm{nm}}=0.96\pm 0.02\;\mathrm{dB}/\mathrm{cm}$ and $\alpha_{625\;\mathrm{nm}}=0.81\pm 0.02\;\mathrm{dB}/\mathrm{cm}$. When subjected to bending, angle-dependent attenuation of approximately $0.02\;\mathrm{dB}{/}^{\circ }$ was observed for both wavelengths within a deflection range of 0–$30^{\circ }$. Since the bending radius was not constant during deflection, this effect is attributed predominantly to increased outcoupling rather than additional absorption.

### 3.2. Sensor Characterization

**Figure 6:**
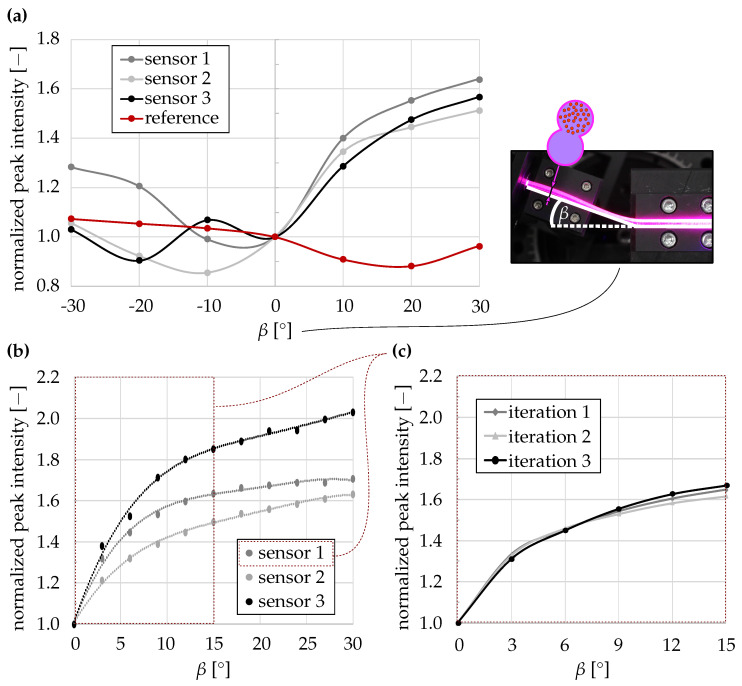
Normalized fluorescence shift-affected intensity ratios of deformation sensors. (**a**) Sensor response for positive and negative bending angles β for three independently fabricated sensors (N = 3) and one fully functionalized reference sensor. (**b**) Mean responses of three sensors for positive bending angles between $0^{\circ }$ and $30^{\circ }$. (**c**) Repeatability of Sensor 1 for three successive measurements with repositioning between $0^{\circ }$ and $15^{\circ }$.

The functionality of the optical deformation sensor was verified by the quantitative measurement of three independently fabricated sensor devices (see Figure 6). A reference sensor with both strands functionalized by QDs exhibited only unspecific signal fluctuations. For the functional sensor, negative bending toward the functionalized strand likewise did not result in a systematic change in the fluorescence shift-affected intensity ratio. In contrast, a clear correlation between the deflection angle β and the normalized peak intensity ratio $R_{norm}$ was observed for positive bending. For all three sensors tested, a distinct increase in the normalized peak intensity ratio was measurable up to a bending angle of $\beta =15^{\circ }$. The characteristic curves of the individual sensors differ in slope and absolute values, indicating geometric or positional variations within the printed structures and resulting in non-interchangeable absolute sensor responses. To assess repeatability, one representative sensor (Sensor 1) was measured repeatedly (N = 3 measurement runs), with the complete repositioning of the sensor strand between runs. The resulting data showed only minor deviations, confirming the high measurement stability and the reproducibility of the optical readout under repeated repositioning.

**Figure 7:**
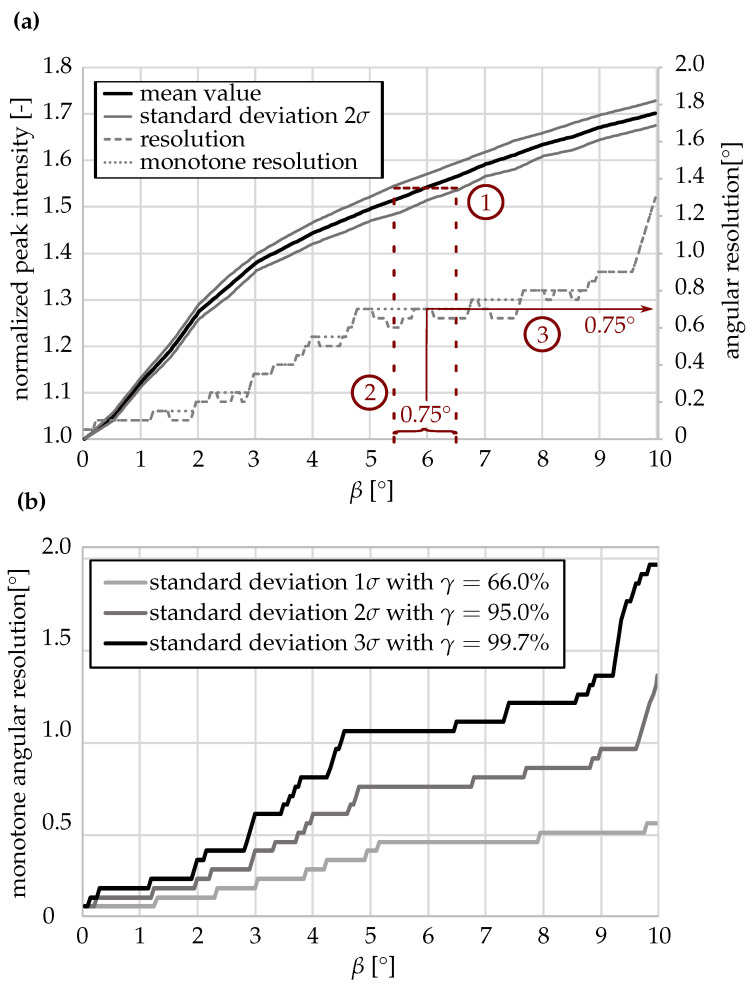
Determination of angular resolution of Sensor 1 for N = 5 independent test runs. (**a**) Mean normalized intensity ratios with $2\sigma_{\mathrm{STD}}$ confidence intervals for 0.5° increments. Evaluation process for the resolution is marked in red. (**b**) Calculated monotone resolution as a function of confidence level γ and deflection β.

To quantify the angular resolution, one of the characterized sensors (Sensor 1) was analyzed with higher angular precision (see Figure 7). This analysis was restricted to a single device (N = 1), as absolute sensor responses were not interchangeable between devices, while the deformation-dependent trend was consistent across all tested sensors. The choice of Sensor 1 was arbitrary within the set of characterized samples and does not imply a preferred or optimized device. Measurements were performed in 0.5° increments within β=0–$10^{\circ }$. This bending angle range was chosen because changes beyond this region were only marginal, and the most sensitive operating range therefore required higher-resolution analysis. The mean values with $1\sigma_{\mathrm{STD}}$, $2\sigma_{\mathrm{STD}}$ and $3\sigma_{\mathrm{STD}}$ confidence intervals were evaluated based on N = 5 repeated measurements at each deflection angle. From the horizontal spacing of the standard deviation bands, the angular resolution was determined for each deflection. At a confidence level of γ=95%, the sensor achieved an angular resolution better than $0.9^{\circ }$ up to $\beta =9.5^{\circ }$. Beyond this angle, the sensitivity decreased significantly due to the saturation of the intensity ratio. A quasilinear sensor response was observed only for small deflections up to approximately $2^{\circ }$, where the measurement accuracy remained below $0.1^{\circ }$. However, this range was not the primary focus of the present investigation.

These results confirm the functionality of the proposed optical sensing framework. While individual sensors exhibit slightly different response characteristics, these deviations can be compensated for through calibration. The repeated measurements demonstrate the high stability and repeatability of the optical signal within the specified range. The effective measurement range of the current sensor design extends from $\beta =0^{\circ }$ to $\beta =9.5^{\circ }$.

## 4. Discussion

Existing optical deformation sensors such as fiber Bragg gratings (FBGs), polymer optical fibers (POFs) or planar photonic structures offer high sensitivity but face distinct limitations in flexibility and functional integration [8,9]. Additively manufactured non-optical sensors, such as conductive elastomer networks, demonstrate integration within soft components but suffer from hysteresis and limited response speeds due to their electrical readout [7,25]. The sensor presented in this work addresses these constraints by employing an optical readout based on quantum dot photoluminescence, which enables inherently fast signal transduction. While the mechanical hysteresis of the silicone structure itself was not quantified in this study, the optical readout mechanism does not introduce additional hysteresis beyond the mechanical response of the material. Through multimaterial embedded printing with an active-mixing printhead, CdSe/CdS quantum dots are locally incorporated into highly transparent silicone waveguides, enabling voxel-level functionalization, high geometric freedom and a mechanically compliant matrix.

Despite these advantages, several technological and methodological challenges remain. The measured attenuation (0.8–1.0 dB/cm at 625 nm and 405 nm) of the printed waveguides exceeds the material-specific minimum of Dowsil™ EI-1184 ($\alpha_{\mathrm{opt}}=0.20\;\mathrm{dB}/\mathrm{cm}$), which is most likely attributed to surface scattering and residual microdefects introduced during the printing process. While the used parameter set for the embedded printing process prevents unwanted effects such as cavity formation, constrictions or ruptures, microscopic inclusions and surface irregularities are presumed to be the dominant sources of optical loss. Consequently, the measured attenuation represents a realistic lower bound for directly printed silicone waveguides, rather than a material-immanent limit. Further refinement of the surface quality and the rheological tuning of the support matrix could narrow the gap to the theoretical transmission limit. Beyond increased optical losses, these surface irregularities and microscopic inclusions also contribute to sensor-to-sensor variability and thus represented a relevant source of measurement uncertainty in our experimental characterization.

From a waveguide design perspective, the present implementation uses air as the cladding medium. This configuration is beneficial for low straight-waveguide attenuation but inherently sensitive to any mechanical contact, which promotes local outcoupling and complicates integration into surrounding structures. Introducing a core-cladding geometry with a tailored refractive index contrast would therefore primarily serve to mitigate contact-induced losses and enable robust embedding into soft robotic components, while at the same time likely increasing the intrinsic attenuation and reducing the number of guided modes. A corresponding core–shell printing strategy—for example, following the coaxial concepts demonstrated by Karyappa et al. [45]—thus requires a trade-off between integration capability and transmission performance and has not yet been realized for the presented sensor architecture.

Regarding sensing performance, the demonstrated detection of angular deformation up to $10^{\circ }$ with a sub-degree resolution confirms the fundamental feasibility of the optical readout concept within the investigated configuration. The quasilinear operating range, defined by the linear regression of the normalized fluorescence-to-excitation ratio as a function of the deflection angle with $R^{2}\geq 0.98$, is given for deflections of $\beta <4^{\circ }$. For larger deflections, the sensor response gradually deviates from global linearity and approaches a monotonic saturation regime. This behavior is attributed to the curvature-induced redistribution of guided modes in the multimode waveguides, where an increasing fraction of modes already interacts with the quantum dot-functionalized region, leading to diminishing changes in the fluorescence-to-excitation ratio with further bending.

Our validation was subject to several experimental constraints. First, the characterization was based on a comparatively small number of waveguides and sensor specimens, and only a single nominal sensor geometry was investigated. The observed sensor-to-sensor variations in the calibration curves indicate that the geometric repeatability of the printed strands and the reproducibility of the voxel-wise quantum dot placement are not yet fully controlled. Second, the bending experiments were conducted for one defined bending setup and essentially uniaxial, stationary bending. Other deformation states, such as different radii, superimposed bending modes, torsion or multiaxial cases, were not considered. In particular, the behavior under repeated cyclic bending, other bending directions or spatially varying curvature along the sensor length remains unexplored, although such conditions are expected in soft robotics or wearable applications. Moreover, the sensor response was evaluated for a specific mechanical fixation concept with the waveguides resting in a V-groove. Possible influences of partial contact-induced outcoupling at the support interface on the fluorescence-to-excitation ratio have not been quantified, so that the transferability of the results to other mounting or embedding conditions is limited. Consequently, the present demonstrator is only validated for contact-free operation under well-defined fixation conditions, as implemented in the experimental setup.

From an optical standpoint, the analysis focused on relative attenuation and spectral ratios, while absolute coupling losses at the interfaces between the excitation fiber, printed sensor and detection fiber were not determined separately. As a result, the absolute optical efficiency of the system and its robustness against variations in alignment or facet quality are possible sources of error. Repeated measurements with repositioning indicate that coupling and alignment-related variability is comparatively small and does not introduce significant additional scatter beyond the intrinsic sample-to-sample variations. Furthermore, the signal depends sensitively on the spatial overlap $l_{v}$ between the excitation light and the quantum dot-functionalized region. Non-uniform particle dispersion, the local agglomeration of quantum dots or minor strand misalignment can modify the fluorescence shift-affected intensity ratio and thus affect calibration and repeatability. These effects therefore constitute an additional material- and geometry-related error source that affects the calibration stability and repeatability of the sensor response. Improving the sensing performance will require optimized strand geometries, improved optical alignment or multi-QD configurations. A further trade-off arises between the fluorescence yield and optical transmission: increasing the quantum dot concentration enhances the signal intensity but simultaneously increases absorption and scattering losses.

Overall, the presented work demonstrates that embedded printing provides a viable route toward optically functionalized elastomeric sensors, combining mechanical compliance with photonic functionality. At the same time, the limited sample size, the restricted set of investigated bending states and the yet-to-be-developed strategies for robust integration into soft robotic structures outline the current methodological boundaries of this first demonstrator and indicate clear opportunities for further development. These aspects, together with the remaining challenges of attenuation, optical confinement and multiaxial sensing, define concrete directions for further optimization, which are addressed in the subsequent section.

## 5. Conclusions and Outlook

In this study, an optical deformation sensor fabricated by embedded printing was demonstrated. Two parallel multimode silicone waveguides—one locally functionalized with CdSe/CdS quantum dots—form the sensing structure. Mechanical deformation modifies the internal light distribution, thereby changing the interaction between guided light and the functionalized strand. The resulting fluorescence shift-affected intensity ratio provides a deformation-dependent signal that allows the detection of angular deflections of up to 9.5° with a sub-degree (<0.9°) resolution at a 2σ confidence level. Both the excitation light and the fluorescence are guided to the endfacet of the waveguide, where a wavelength-selective detector separates and records their respective spectral contributions. In future implementations, this functionality will be realized using optical filters and two photodiodes instead of a monochromator, enabling compact, low-cost and fully integrable readout electronics. The demonstrated system thus validates the underlying optical principle and confirms the feasibility of functional integration by embedded printing.

The presented method showcases the potential of additive manufacturing for integrated photonics in soft matter systems. Beyond this proof of concept, future research will target the reduction of optical attenuation by refining the surface quality, the implementation of core–shell geometries for better integrability and multiaxis or spatially resolved sensing configurations to capture complex deformation states. These multiaxial and spatially resolved deformation sensors can be realized by integrating multiple quantum dot systems with distinct emission characteristics, either distributed across the sensor cross-section or localized at defined positions along the waveguide length. The ability to print voxel-precise distributions of nanomaterials opens up pathways toward soft robotic actuators with embedded feedback, functionalized wearable devices and large-area, conformal sensing surfaces.

## Data Availability

All raw data are freely available. Attenuation experiments: https://doi.org/10.25835/ai8koh7u. Sensor experiments: https://doi.org/10.25835/khimutfn.
